# Reproductive performance of indigenous Lao pigs reared by small-scale farmers in northern provinces of Laos

**DOI:** 10.5194/aab-64-365-2021

**Published:** 2021-09-09

**Authors:** Somsy Xayalath, Gabriella Novotni-Dankó, Péter Balogh, Klaus-Peter Brüssow, József Rátky

**Affiliations:** 1 Doctoral School of Animal Husbandry, University of Debrecen, Böszörményi Street 138, 4032 Debrecen, Hungary; 2 Institute of Animal Science, Biotechnology and Nature Conservation, Faculty of Agricultural and Food Sciences and Environmental Management, University of Debrecen, Böszörményi Street 138, 4032 Debrecen, Hungary; 3 Department of Statistics and Methodology, Institute of Statistics and Methodology, Faculty of Economics and Business, University of Debrecen, Böszörményi Street 138, 4032 Debrecen, Hungary; 4 Centre of Veterinary Sciences, Nicolaus Copernicus University, Toruń, Poland; 5 Department of Obstetrics and Food Animal Medicine Clinic, University of Veterinary Medi-cine Budapest, Budapest, Hungary

## Abstract

Indigenous pigs are essential domestic animals for rural life and meat supply in Laos, especially for ethnic people in remote areas. Northern provinces have the most numerous indigenous pig populations, i.e. covering 84 % of the total pig population. This study was conducted in northern Laos, where 164 pig-raising households, 325 sows and 1246 piglets were included. The study aimed to observe the general trend of change in indigenous pig utilization and the altered reproductive performance regarding village location and rearing systems. The semi-structured questionnaires were a key tool for gathering data required through personal interviews and field observations. Two types of indigenous Lao pig breeds (locally named Moo Lath and Moo Hmong) were found in study areas. The village locations were not influencing on reproductive performance of indigenous Lao pigs. Larger litter size and birth weight (P<0.004–0.000) was found in the second cluster (15 to 30 km away from downtown) with an average of 8.24 heads and 0.88 kg, while the first (<15 km) and third (>30 km) clusters had 7.72 versus 7.12 heads, and 0.70 versus 0.63 kg, respectively. Conversely, the second cluster had lower litter per year (P<0.001) by 1.04, compared to 1.38 for the first and third clusters. The free-scavenging rearing system (FRS) had a higher litter size (8.5) than the confinement (CRS) and semi-scavenge (SRS) rearing system (7.36 versus 7.54). The FRS had a marginally smaller litter per year (0.87) that differed from the CRS and SRS (1.45 and 1.41). The CRS had a shorter suckling period (2.38 months) with a lower weaning weight (6.74 kg), while the FRS and SRS had longer (2.72 versus 2.8 months) and higher weaning weight (7.76 and 7.57 kg). The mortality before weaning was 15 %, and no difference was found related to the villages' location or rearing systems (P>0.070 versus 0.839). Around 56 % of the piglet's deaths were due to poor management that caused piglets to be crushed/injured by sow or starvation. More than 54 % of farmers did not keep sows in pens before the farrowing, and 53 % of sows gave birth near forests. In conclusion, the village locations and rearing systems did not influence the reproductive performance of indigenous pigs in northern Laos. However, pre- and post-farrowing management had a strong effect on it. During the whole study, we took into consideration the successful example of Hungarian Mangalica pig, which could find a proper new role in the global premium markets. Our results suggest that similar complex semi-intensive farm operations as indigenous Mangalica pig farms in Hungary should be a great option for introducing and adapting to improve indigenous pig performance in Laos.

## 1 Introduction

Indigenous pig breeds are still dominant in Laos, which covered more than 91 % of the total pig population (3.1 million) in 2014. Most of them were raised by smallholder farmers, in particular, in rural or mountainous areas (Keonouchanh, 2018). Indigenous pigs not only contributed to meat consumption and primary income for households (Holt et al., 2019; Okello, 2017), but also they were also important to the traditional activity performance of farmers, especially ethnic people living in remote areas of Laos (Keonouchanh et al., 2014). In a similar report of Xayalath et al. (2020), indigenous pigs play an important role in the supply of meat and contribute between 9 %–14 % of the annual household income in the rural areas of Laos. It could be said that the ethnic people (Lao-Tai, Mon-Khmer, Hmong-Mien, Tibeto-Berman) living in the mountainous areas of Laos, especially in the north, could not abandon indigenous pig raising (Phengsavanh et al., 2011). In 2019, Laos counted a pig population of about 4.1 million, including 1.4 million in the north, 1.2 million in the central region, and 1.5 million in the south (MAF, 2020). Lao government has been active in encouraging farmers to move from conventional production to farming production, which could both control quality and increase pork production to meet pork consumers' needs (MAF, 2015). However, the southern part had the largest number of pigs (with more commercial pig farms). Still, the northern region also had the second largest pig population with an enormous number of indigenous pigs, almost 85 % of the total pig population. In northern Laos, more than 60 % or some areas (Phongsaly Province), more than 90 % of the total households owned at least one indigenous pig (Epprech et al., 2018). Unfortunately, since 2016, not only in Laos, the number of indigenous pigs has decreased. Because of the African swine fever (ASF) disease and other factors, it has rapidly declined in all Association of Southeast Asian Nations (ASEAN) countries as well. However, indigenous pigs produce delicious and healthy pork, and the new market options should be found for the survival of these breeds and for the poverty alleviation in rural areas. Indigenous pig production in Laos is facing two main chronic problems: sows give a small number of piglets per litter and poor farm management, causing various negative impacts on pig performance. This includes only about 5–8 piglets per litter with a few survival piglets at the time of weaning (mortality rate 20; 50 %) and poor growth rate (Chittavong et al., 2012; Phengsavanh et al., 2010) with an average daily gain less than 120 $g\;d^{-1}$, and high fat rate about 65 %–70 % (Keonouchanh and Dengkhounxay, 2017). Such problems, including the quality of productive and reproductive performance, are still a concrete problem that blocks the progression of improving indigenous pig performance in Laos. These issues are waiting to be addressed, especially primarily housing and feeding technology, animal health care, and better marketing options. The fruitful experience of the Mangalica indigenous pig farm operation in Hungary, Black Iberian pigs, and other developed European indigenous pig breeds could be the best example for the world's indigenous pig development model.

We need to find out all the holes in the development of indigenous pigs and find the options of the better practices of Hungarian Mangalica pig farms in Hungary, to be complied with and adopted or recommended to improve the quality of indigenous pig performance for farmers in Laos. This study was conducted with two main objectives. The first was to study the general trend of the indigenous pig population and compare the reproductive performance of indigenous pigs based on clustering villages and rearing systems. We also indicated two hypotheses that indigenous pigs raised by farmers who live near downtown would have better reproductive performance parameters than those raised by farmers who live far away from downtown because of easier access to feedstuffs and other facilities. Another hypothesis was that the indigenous sows raised in the confinement rearing system would have better reproductive performance parameters than the other two raising systems (free-scavenge and semi-scavenge rearing systems), as farmers would provide them with better feedstuffs and other practices.

**Figure 1:**
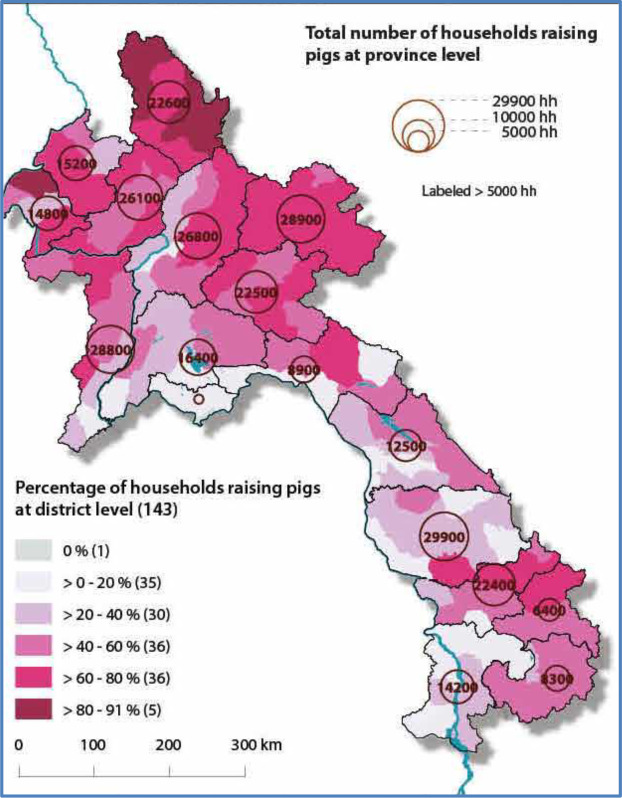
Map of the survey area; Epprecht et al. (2018), CC BY 4.0.

## 2 Materials and methods

### 2.1 Study area

This study was conducted in northern provinces of Laos, where there were densely populated indigenous pig-raising households. This involved about 60 % to 90 % of the whole households engaged in indigenous pig farming in the region (Epprecht et al., 2018, CC BY 4.0; Fig. 1). This region is a mountainous area, home to different ethnic groups such as Lao-Tai, Mon-Khmer, Hmong-Mien, and Tibeto-Berman (Messerli et al., 2008), who consider indigenous pigs part of their livelihood. In northern provinces, most pig raising was based on traditional systems such as simple confinement, free scavenging, and semi-scavenging. Most farmers fed their pigs based on feed produced from agricultural production, i.e. maize, cassava, rice brand, and forest vegetation (Phengsavanh et al., 2010).

### 2.2 Sampling procedures and sample size

Five provinces (Phongsaly, Oudomxay, Xayaboury, Louangprbang, and Houaphanh) out of seven provinces in northern Laos were selected to participate. Phongsaly had the highest number of pig-raising households, ranging about 80 %–91 %. While other provinces had between 60 % and 80 % (Epprecht et al., 2018, CC BY 4.0; Fig. 1). One or two districts were selected from each province. Each district selected one to three villages to represent the survey, based on discussion with each livestock unit of DAFO (District agriculture and forestry office). Out of 8 districts, 164 families of pig-raising households were selected to participate in the survey covering 325 sows and 1246 piglets. A household with at least one indigenous sow with one farrowing experience, and who volunteered to respond to the questions, was considered to qualify for the sampling.

### 2.3 Methods of data collection and measurement

The semi-structured questionnaires were a pivotal tool to collect primary data from pig-raising households based on individual interviews. The pig population's statistics for the last 5 years were collected through the livestock section of each PAFO (province agriculture and forestry office) of all northern provinces of Laos. These data were used to analyse the indigenous pig population trend in northern provinces of Laos (Table 1). The general pig production data were discussed with the respective DAFO livestock unit team of each selected district. The personal interviews using the prepared semi-structured questionnaires were carried out to obtain the primary data of the reproductive performance of indigenous sows. In order to get different data on reproductive performance parameters, raising system, managements, problems, and causes of piglet mortality, the target villages were classified into three clusters based on *kilometres* away from downtown. This is slightly different from Phengsavanh et al. (2010) studied, which was clustered based on *hours*. It is very similar to the methods' study of Valle Zárate et al. (2010), which was carried out in the northwest of Vietnam. The first cluster was less than 15 km away from downtown, the second was between 15 and 30 km away, and the third target was more than 30 km far away from downtown. A total of 164 pig-raising households participated in the survey, including 54 households less than 15 km, 41 households between 15 and 30 km, and 69 households over 30 km away from downtown.

### 2.4 Statistical analysis

The collected data were entered into Microsoft Excel version 2010 and stored before analysis. Descriptive statistics were used for pig population trends presented by graphs and bar charts, including the percentages and differences in breeding management, farrowing management, and the causes of piglet death. These data were analysed using Microsoft Excel 2010. One-way ANOVA and Tukey's post hoc test of the SPSS statistic version 26 (2019) was used to check for the significant differences in the mean values of reproductive performance parameters, in particular age of obtained puberty, age of first mating, litter size, birth weight, suckling period, weaning weight, the mortality of piglets, and the lifespan of sows and boars. The significant level of 0.05 (P<0.05) was used for the different reproductive performance parameters of piglets born from indigenous sows in the various locations, rearing systems, and other parameters. Bivariate correlation Pearson was used for analysing the correlation coefficients among reproductive traits.

## 3 Results

### 3.1 General trend of indigenous pig population and its production

However, the indigenous pig population's overall trend in the study areas was a decline from 2015. Still, at the time of the study, the indigenous pig population in northern provinces of Laos was high at 84.5 %. Table 1 showed that the percentage of indigenous pig population was consecutively decreased from 93.51 % in 2016 to 84.57 % in 2019. Regarding the livestock section of each PAFO reported, there were some new investments on commercial pig farms of Chinese companies in larger provinces in the north of Laos such as Louangnamtha, Oudomxay, and Louangprang from the second half of 2016. In addition, all PAFOs and DAFOs reported that ASF was an epidemic disease that killed a large number of indigenous pigs raised by small-scale farmers in the north of Laos. It might be a result of the indigenous pig population decreasing by almost 5 % on average in 2019 compared to 2018. Some provinces such as Oudomxay declined by almost 15 %, and Louangprabang Province was around 7 % on average (Table 1).

**Table 1:** The trend of indigenous pig population in northern Laos from 2015–2019 (thousand heads).

	2015			2016			2017			2018			2019		
	TPP	NPP	%	TPP	NPP	%	TPP	NPP	%	TPP	NPP	%	TPP	NPP	%
PhP	231	230.5	99.78	263	262	99.61	240	238	99.16	249	246	98.79	194	191	98.45
LNP	109	103	94.49	110	105	95.45	132	119	90.15	130	117	90.00	137	121	88.32
OP	124	99	79.83	136	109	80.14	119	83	69.74	134	80	59.70	131	59	45.03
BP	75	71	94.66	85	81	95.29	89	85	95.50	88	79	89.77	86	77	89.53
LPP	232	197	84.91	241	228	94.60	248	234	94.35	248	239	96.37	278	250	89.92
HP	162	161.5	99.67	143	142	99.30	153	152	99.34	163	159	97.54	133	130	97.74
XP	146	124	84.93	148	126	85.13	167	133	79.64	173	137	79.19	182	137	75.27
GT	1079	986	91.40	1126	1053	93.51	1148	1044	90.93	1185	1057	89.19	1141	965	84.57

TPP: total pig population; NPP: native pig population; %: percentage of native pig population; PhP: Phongsaly Province; LNP: Louangnamtha Province; OP: Oudomxay Province; BP: Bokeo Province; LPP: Louangprabang Province; HP: Houaphanh Province; XP: Xayaboury Province; GT: grand total of pig population.

### 3.2 Overall of indigenous pig reproductive performance in northern provinces of Laos

**Figure 2:**
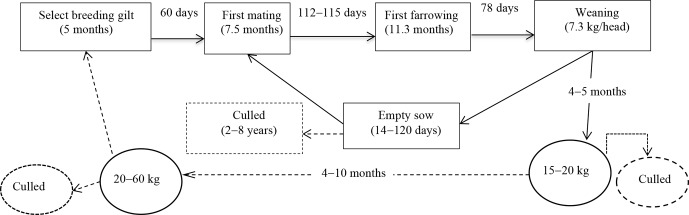
Indigenous pig reproductive and productive cycle. AFM: age of first mating (month).

The average age for obtaining puberty of indigenous gilts was around 7.03±1.35 months in the survey areas. Still, there was also a large difference between households ranging from 4 to 11 months, depending on raising conditions. Most farmers let their gilts breed as soon as they have their first oestrus cycle (87.8 % of total households), so the first mating was around 7.5±1.43 months, with a range of 5–12 months. The first farrowing was between 8.8 and 15.8 months, or an average of approximately 11.03±1.43 months (Fig. 2). The average litter size was about 7.60±1.77 heads, ranging from 4 to 11 heads per litter. Overall, the sow would give approximately 1.30±0.53 litters per year, ranging between 0.7 to 2 litters per year. The average birth weight of the new born piglets had around 0.72±0.23 kg. However, it had a long suckling period of about 2.62±0.79 months, but the weaning weight was only 7.30±2.12 kg on average. The sow had the mean interval oestrus after weaning around 48.34±27 d. Farmers used their sows for 2 to 8 years, approximately 4.66±1.39 years on average.

There are significant differences in several reproductive performance parameters based on villages' cluster locations and rearing systems (Tables 2 and 3). In particular, the second cluster (SC=15 to 30 km) had the largest litter size and birth weight (P<0.004 and 0.001) with an average of about 8.24 heads and 0.88 kg. On the other hand, the first (FC= less than 15 km) and third (ThC= over 30 km) clusters had around 7.72 heads and 7.12 heads, as well as 0.70 and 0.63 kg, respectively. The SC was found to have the lowest litter per year (P<0.001), averaging 1.04, while the FC and ThC were about 1.38. A long suckling period with a corresponding better weaning weight (P<0.021) was found in the ThC (2.93 months with 7.83 kg), while the FC and SC were 2.36 versus 2.43 months, and 6.86 versus 6.97 kg. This study found a different lifespan (P<0.001) of the sows in the three clusters. The SC had the longest use of sows with around 5.45 years, while the FC and ThC were approximately 3.98 and 4.72 years, respectively.

**Table 2:** Comparison on reproductive performance of indigenous pigs based on village locations.

Parameters	Distance of village location (km)			Mean ± SD	P value	SEM
	<15	15 to 30	>30			
Number of households	54	41	69			
Age of gilt to be selected as breeding gilt, month	5.63${}^{a}$	5.41${}^{a}$	5.13${}^{a}$	5.37±1.82	0.318	0.14
Age of puberty, month	6.85${}^{a}$	7.00${}^{a}$	7.18${}^{a}$	7.02±1.34	0.403	0.10
Age of first mating, month	7.42${}^{a}$	7.34${}^{a}$	7.65${}^{a}$	7.50±1.43	0.474	0.11
Age of first farrowing, month	11.22${}^{a}$	11.14${}^{a}$	11.45${}^{a}$	11.30±1.43	0.474	0.11
Litter size, head	7.72${}^{a}$	8.24${}^{b}$	7.12${}^{a}$	7.60±1.76	0.004	0.13
Litter per year, time	1.38${}^{a}$	1.04${}^{b}$	1.38${}^{a}$	1.29±0.52	0.001	0.04
Birth weight, kg	0.70${}^{a}$	0.88${}^{b}$	0.63${}^{a}$	0.72±0.22	0.000	0.01
Suckling period, month	2.36${}^{a}$	2.43${}^{a}$	2.93${}^{b}$	2.62±0.79	0.000	0.06
Weaning weight, kg	6.86${}^{a}$	6.97${}^{a}$	7.83${}^{b}$	7.29±2.12	0.021	0.16
Mortality before weaning, head	1.33${}^{a}$	1.08${}^{a}$	1.10${}^{a}$	1.17±0.94	0.305	0.07
Stillbirth, head	0.19${}^{a}$	0.13${}^{a}$	0.10${}^{a}$	0.14±0.40	0.450	0.30
Interval oestrus after weaning, d	49${}^{a}$	47${}^{a}$	48${}^{a}$	48±27	0.963	2.11

${}^{a,b}$ Means in the same row with different superscript differ significantly (p<0.05). <15: villages with less than 15 km away from the city centre. 15 to 30 km: villages with locations between 15–30 km away from the city centre. >30: villages with more than 30 km away from the city centre.

The free-scavenging rearing system (FRS) had a larger litter size, birth weight, weaning weight, and longer lifespan of sows (Table 3) than the other two rearing stems. There was a difference (P<0.001) in FRS with approximately 8.5 piglets per litter, which was higher than the confinement (CRS) and semi-scavenge (SRS) rearing systems with an average of about 7.36 and 7.54. A slightly low litter per year with around 0.87 differs (P<0.001) from the CRS and SRS, which had around 1.45 versus 1.41. The differences in litter size and the number of farrowing per year could result from different breeds (Moo Lath, Moo Hmong, and crossbred among them). On the other hand, the weaning weight might have had an effect on the lactation period, as the CRS had a shorter period (2.38 months) with the consequence of a lower weaning weight (6.74 kg). The FRS and SRS had a longer period of 2.72 and 2.8 months and had a higher weaning weight of about 7.76 and 7.57 kg, respectively. The duration of farmers using their sows and boars among their rearing systems was another point to note when the free-scavenge system had a longer period of using sows (5.8 years) and a second longer period (3.5 years) of use of their boars.

**Table 3:** Comparison of the reproductive performance of indigenous pigs based on rearing systems.

Parameters	Pig rearing systems			SEM
	Confinement	Free scavenging	Semi-scavenging	
Number of households	64	41	59	
Age of gilt to be selected as breeding gilt, month	4.93${}^{a}$	5.24${}^{a}$	5.92${}^{b}$	0.14
Age of puberty, month	6.83${}^{a}$	7.09${}^{a}$	9.18${}^{a}$	0.10
Age of first mating, month	7.49${}^{a}$	7.46${}^{a}$	7.54${}^{a}$	0.11
Age of first farrowing, month	11.29${}^{a}$	11.26${}^{a}$	11.34${}^{a}$	0.11
Litter size, head	7.36${}^{a}$	8.50${}^{b}$	7.22${}^{a}$	0.13
Litter per year	1.45${}^{a}$	0.87${}^{b}$	1.41${}^{a}$	0.04
Birth weight, kg	0.70${}^{a}$	0.79${}^{a}$	0.69${}^{b}$	0.01
Suckling period, month	2.38${}^{a}$	2.72${}^{b}$	2.80${}^{b}$	0.06
Weaning weight, kg	6.74${}^{a}$	7.76${}^{b}$	7.57${}^{b}$	0.16
Mortality before weaning, head	1.17${}^{a}$	1.11${}^{a}$	1.22${}^{a}$	0.07
Stillborn, head	0.20${}^{a}$	0.06${}^{a}$	0.12${}^{a}$	0.03
Interval oestrus after weaning, d	46${}^{a}$	48${}^{a}$	50${}^{a}$	2.11
Lifespan of sows, year	4.03${}^{a}$	5.80${}^{b}$	5.54${}^{c}$	0.10
Lifespan of boars, year	3.81${}^{a}$	3.50${}^{a}$	2.76${}^{b}$	0.10

${}^{a,b,c}$ Means in the same row with different superscript differ significantly (p<0.05).

**Figure 3:**
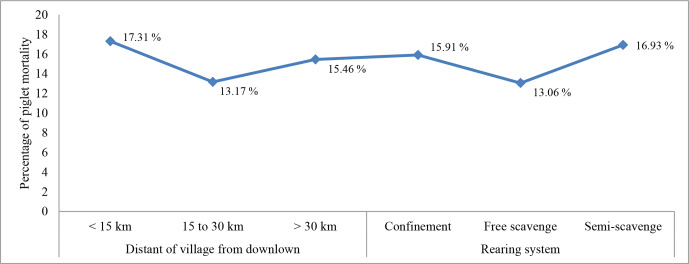
Comparison of piglet mortality based on village clusters and rearing systems.

The piglets' average mortality rate before weaning was around 15.45 % and did not differ between village locations or rearing systems (P>0.305 versus 0.839). However, the free-scavenging rearing system had the lowest mortality with around 13 % compared to confinement, and semi-scavenging was 15.91 % and 16.93 %, respectively (Fig. 3). Approximately 56 % of the piglet deaths were due to inadequate management during farrowing and lactation, which caused piglets to be crushed by their mother and starved. More than 54.26 % of farmers did not keep their sows in pen before the farrowing day, and more than 53.66 % of farmers allowed their sows to give birth to piglets in the nearby forest. This may be one of the reasons for the death of piglets before weaning time.

Table 4 shows the age of puberty estimated strongly positively correlated to the age of first mating and the age of first farrowing. In contrast, litter per year and birth weight are highly negatively correlated with suckling period and weaning weight.

**Table 4:** Correlation coefficients among reproductive traits of indigenous pigs in northern Laos.

Parameters	AP	AFM	AFF	LS	LPY	BW	SP	WW	MBW	IEW
AP	1.000	0.817${}^{**}$	0.817${}^{**}$	0.053	0.042	-0.010	0.048	0.015	0.137	-0.031
AFM		1.000	1.000${}^{**}$	0.043	0.039	-0.030	-0.036	-0.078	0.188${}^{*}$	0.118
AFF			1.000	0.043	0.039	-0.030	-0.036	-0.078	0.188${}^{*}$	0.118
LS				1.000	0.021	0.232${}^{**}$	-0.144	-0.015	0.129	-0.124
LPY					1.000	0.142	$-0.228^{**}$	$-0.182^{*}$	0.126	$-0.353^{**}$
BW						1.000	-0.224${}^{**}$	$-0.171^{*}$	-0.26	-0.027
SP							1.000	0.554${}^{**}$	-0.034	-0.090
WW								1.000	-0.138	-0.127
MBW									1.000	0.010
IEW										1.000

${}^{**}$ correlation is significant at the 0.01 level (two-tailed). ${}^{*}$ correlation is significant at the 0.05 level (two-tailed); AP: age of puberty; AFM: age at first mating; AFF: age at first farrowing; LS: litter size; LPY: litter per year; BW: birth weight; SP: suckling period; WW: weaning weight; MBW: mortality before weaning; IEW: interval estrous after weaning.

## 4 Discussion

In the study areas, indigenous pig production was mainly based on traditional rearing systems such as simple confinement, free scavenging, and semi-scavenging and found to be around 39 %, 25 %, and 36 %, respectively. The pig rearing systems depended on the seasons of agricultural production and the village regulations. Most of the villages in the study areas are now striving hard to build and lead their villages to meet the criteria of becoming a sanitized and developed village, with respect to three constructive levels of national development strategy (Central committee party office, 2012). Keeping animals in pens, especially pigs, is also one of the criteria that every village has to consider and follow as a wide-range regulation in many areas. Pig raising as a semi-scavenging rearing system usually comes in two forms. In the first form, farmers kept their pigs in pens only in the night-time after they fed them in the evening. During the daytime and otherwise, they would keep their pigs in pens only during the main crop production season and leave them in free-scavenging mode again after harvest. This is very similar to earlier Hungarian farmers in the villages who kept their Mangalica pigs indoors only in the night-time, and they grazed all their pigs in the communal pasture (Egerszegi et al., 2018).

Pig housing, nutrition condition, and proper selection of both gilts and boars are three main issues to consider for improving indigenous Lao pig breeds' current productive and reproductive performance in northern Laos. Egerszegi et al. (2018) summarized the past and current status of Hungarian Mangalica pig with special regard to breeding and reproductive performance research: (1) select replacement gilts, large litter, and at least 10 functional teats; (2) stimulate gilts and weaned sows to come into heat at a similar time and get piglets in the same batch; (3) improve housing and nutrition in case of intensive breeding; and (4) improve feeding of sows bred intensively and culling after sows have achieved her six–seventh farrowing. These successful development tracts should be introduced to improve the quality of indigenous Lao pigs.

Two different types of local pig breeds – i.e. Moo Lath and Moo Hmong – were found in the study areas. These two types were commonly found in northern Laos. These two breeds were different in the growth rate and reproductive performance, especially litter size of 7–8 versus 7–10 (Keonouchanh et al., 2011). However, no evidence on genetic information was found that described the difference between these two types of pig breeds, but their phenotypes were so different among them (Wilson, 2007). Due to the differences between types of breeds, this may be one way of explaining the difference in litter size and birth weight (Gokuldas et al., 2015). It was found that there was no difference between FC (<15 km) and ThC (>30 km), but the SC (15 to 30 km) had a high litter size and birth weight. However, the SC had a slightly low litter size per year. It may not be surprising that the average weaning weight was higher in the ThC (7.83 kg) than FC (6.86 kg) and SC (6.97 kg). This might because of having a longer period of suckling (2.93 months) compared to FC (2.36 months) and SC (2.43 months). Wilson (2007) also reported that Moolath pigs reached puberty at around 6 months of age, piglets weaned at 2–3 months at around 7–8 kg, and sows had about 1.5 litters per year. Therefore, it was hypothesized that the farmers did not consider using the community feedstuff services and other facilities available in the nearby cities. Most pig farmers in northern Laos mainly used locally available feed by-products from agricultural production and seasonal forest vegetables to feed their pigs (Phengsavanh et al., 2011). This finding slightly differed from the study of Phengsavanh et al. (2010), which concluded that the weight gain of fattening pigs raised by farmers in northern provinces of Laos, who lived near the city centre, was better than that of farmers who lived far from the city centre.

This study's findings may also differ from other theories and research where it was found that many parameters of indigenous pig reproductive performance in the free scavenging were better than the confinement and semi-scavenging rearing systems, especially the litter size, birth weight, and weaning weight. It might be hard to explain with the right reason; again it could be assumed that feeding and scavenging could be one option to consider what made them different. There was no difference in feeding among the three different rearing groups. Farmers always fed their pigs once in the morning and another once in the evening with available local feed resources such as cassava roots, rice bran, and kitchen waste produced from agricultural production (Huynh et al., 2006). Due to the smallholders, farmers could not buy high-quality feedstuffs or completed feed for their pigs (Stur et al., 2002). The pigs in the free-scavenge rearing system were able to consume, including scavenging, and were fed by their owners in the morning and evening.

Almost 51 % of farmers reported that their sows gave birth during the main agricultural production season between June and September. Around 24 % gave birth to the piglets after the harvesting season between October and December and about 25 % during the non-agricultural production season. For most wild boars and earliers Hungarian Mangalica pigs the heat and mating occurred in the autumn and farrowing 4 months later in the spring, and sometimes a second farrowing might have again in August and September (Webster, 2011; Egerszegi et al., 2003, 2018). It is also hard to accurately describe the difference (P<0.038) in the mean of litter per year of the sows, which farrowed during the primary crop production, after harvesting, and non-agricultural production seasons at 1.40, 1.18, and 1.20, respectively. However, there was no difference (P>0.050) in the mean of the litter size, birth weight, weaning weight, and mortality of piglets before weaning for the sows farrowed during main crop production, after harvesting, and non-agricultural production season. But it was found that the piglets farrowed during the main crop production and after harvesting season had a slightly higher birth weight (0.73 versus 0.77 kg) than those born during the non-agricultural production season (0.68 kg). Conversely, piglets were farrowed during main crop production and after harvesting season had a slightly high mean piglet mortality before weaning (1.23 versus 1.14 heads per litter) compared to piglets born during the non-agricultural production season (1.10 heads per litter). It was assumed this was because of improper management during farrowing. Besides, these two seasons, farmers always kept their sows in pens, which could make it easy for sows to crush their piglets.

The mortality rate (15.45 %) was lower than the study of Phengsavanh et al. (2010) with around 50 %, which was carried out in the same region, and the study of Chittavong et al. (2012) with around 20 %, which was conducted in the central provinces of Laos. It could be considered that the piglet mortality might be high for the early weaning due to new transit and improper feeding by farmers. Based on the early weaning period, one of the key aspects to consider for the piglet survival and growth rate is that new transit could increase more stress, and new feed could be harmful to the new transit piglets (Campbell et al., 2013). It was interesting to note that this study found that the FRS had the lowest piglet mortality rate (13 %), compared to CRS (15.91 %) and SRS (16.93 %). The statistics are presented in Fig. 3. It was hypothesized that some farmers might lose the monitoring of their sows for the first three days after farrowing. All households from the FRS group responded that they left their sows to give birth in the nearby forest. Some of them did not even know that their sows had already given birth to the piglets until the sows brought their piglets back home. Again, it was assumed that farmers might not know how many piglets had died before they had been found. Before weaning, most piglet mortality always occurred during the first three days after farrowing, due to weakness, lack of immunity, and the fact that piglets can be easily crushed by their mother (Deviller et al., 2011).

## 5 Conclusions

However, the population of indigenous pigs has decreased year by year due to Lao consumers' eating preferences. They increasingly prefer lean (low fat) pork that is provided by exotic pig breeds rather than fatty pork that is characteristic of indigenous Lao pig breeds. Still, indigenous pigs are a key component of meat consumption in Laos. Most farmers raise their pigs mainly under traditional practices, so proximity to downtown or facility services is not crucial for reproductive performance of indigenous Lao pig breeds. In general, the pig rearing systems also do not affect the reproductive performance of indigenous Lao pigs. This study showed that the free-scavenging rearing system had a marginally better litter size and birth weight and had the lowest piglet mortality rate compared with confinement and semi-scavenging rearing systems. Most previous research stated that the free-scavenging rearing system was poor in every parameter of productive and reproductive performance of indigenous pigs compared with other rearing systems. The local community services (e.g. providing feedstuff) and rearing systems have no strong influence on the farmers with special regards to reproductive performance of indigenous Lao pigs. However, proper farm management might have a major impact on overall productivity. Actually, most Lao farmers still raise their indigenous pigs in a traditional extensive system and do not operate their farms in a business manner. Therefore, the combination of the extensive and intensive model of Hungarian Mangalica pig breeding and production system might be a positive option to introduce and adapt to improve indigenous Lao pig performance. Based on the present study, we can conclude that Lao farmers might be able to improve housing and feeding methods and to develop relevant reproductive management of indigenous pigs with the goal of increasing the number of surviving market-weight offspring.

## Data Availability

The data sets are available upon request from the corresponding author.
